# Orphan medical devices: addressing the regulatory and access gaps in the EU and US

**DOI:** 10.3389/fpubh.2025.1729821

**Published:** 2026-01-14

**Authors:** Sanae Akodad, Baptiste Haon, Lise Rochaix, Hilde Stevens

**Affiliations:** 1 Institute for Interdisciplinary Innovation in Healthcare (I3h), Faculté de Médecine, Solvay Brussels School of Economics and Management, Université Libre de Bruxelles (ULB), Brussels, Belgium; 2 Paris School of Economics – Hospinnomics, Paris, France; 3 Université Lyon 2, GATE UMR 5824, Lyon, France

**Keywords:** access pathways, adaptive evidentiary pathways, argus ii, Berlin heart EXCOR, economic incentives, evidentiary standards, exit-insurance schemes, harmonized HTA framework

## Abstract

**Background:**

Orphan medical devices remain the blind spot of rare disease policy. While the United States has recognized them since 1990 through the Humanitarian Use Device (HUD) and Humanitarian Device Exemption (HDE) framework, the European Union only introduced a non-binding definition in 2024 (MDCG 2024–10), revealing a deeper asymmetry: flexibility is codified in the US but interpreted in the EU.

**Methods:**

We conducted a document-based policy analysis (1990–2025) and examined legislation, regulatory guidance, and academic literature to compare EU and US frameworks across evidentiary standards, access pathways, economic incentives, and reimbursement-post-market alignment. Three illustrative case studies (Berlin Heart EXCOR, Argus II, and the Medtronic Melody valve) were used to assess real-world consequences for access, sustainability, and patient continuity of care.

**Results:**

The EU’s Medical Devices Regulation 2017/745 lacks a legal orphan-device designation, dedicated incentives, or a harmonized HTA framework. Access remains fragmented, and recertification under the MDR threatens supply continuity. The US HUD/HDE pathway codifies flexibility through a “probable benefit” standard under IRB supervision and an annual distribution cap, though financial sustainability remains limited by profit restrictions and inconsistent reimbursement. Case comparisons reveal: (i) earlier EU access without post-market safeguards (Argus II); (ii) US capacity to transition from humanitarian to full approval when incentives align (Melody); and (iii) vulnerability of SMEs to compliance costs (EXCOR).

**Conclusion:**

Addressing this policy vacuum requires an inflection point equivalent to the 1983 Orphan Drug Act, one that redefines how innovation for the few is valued. Five policy pillars emerge: a legal designation, adaptive evidentiary pathways, sustainable incentives (e.g., pooled procurement), integrated reimbursement mechanisms, and mandatory continuity-of-access or “exit-insurance” schemes. Without such reform, essential technologies for rare diseases will remain trapped between regulatory rigidity and market abandonment. This policy paper highlights structural regulatory gaps and proposes actionable reforms to ensure equitable access to orphan medical devices.

## 1 Introduction

Medical innovation for rare diseases has undergone a profound transformation over the past three decades, with the development of orphan drugs increasingly supported by dedicated regulatory frameworks, financial incentives, and structured early access pathways (1, 2). By contrast, orphan medical devices intended for the diagnosis, prevention, or treatment of conditions affecting extremely small patient populations remain under-recognized, under-incentivized, and structurally disadvantaged across global regulatory systems (3). These disparities are especially pronounced in the European Union (EU), where the absence of a formal legal designation for orphan devices has historically impeded both development and deployment, despite growing demand from clinicians and patients for device-based solutions in rare disease contexts (4).

The consequences are both clinical and systemic. While many rare conditions have no pharmacological treatment, they may be manageable through novel assistive, diagnostic, or implantable devices (e.g., pediatric cardiac pumps, neural stimulators, or implantable prostheses). Yet the challenges of developing such devices are magnified by the very features that define rare diseases: small, geographically dispersed populations, ethical constraints in clinical testing, high upfront development costs, and uncertain commercial returns (5, 6). For many developers, particularly academic groups and small to medium-sized enterprises (SMEs), these hurdles are prohibitive without dedicated pathways for expedited regulatory review, evidentiary flexibility, and sustainable reimbursement.

The European regulatory system for medical devices changed profoundly with the implementation of Regulation (EU) 2017/745 (Medical Devices Regulation, MDR), which introduced stricter requirements for clinical evaluation, reinforced post-market clinical follow-up (PMCF), and enhanced vigilance reporting obligations (7). However, the MDR did not establish a designation pathway or dedicated incentives for orphan devices, in contrast to Regulation (EC) No. 141/2000 for medicinal products, which provides a formal orphan designation, protocol assistance, and market exclusivity (8). Instead, a piecemeal and fragmented landscape has persisted, with individual Member States invoking Article 59 derogations (temporary authorizations allowing market access without CE marking in exceptional circumstances) and clinicians relying on custom-made or legacy devices under increasingly stringent conditions. Only in June 2024 did the EU’s Medical Device Coordination Group (MDCG) issue formal guidance (MDCG 2024–10) defining orphan medical devices (limited to conditions affecting not more than 12,000 patients per year in the EU) and outlining acceptable evidentiary standards and post-market commitments (9). Although this represents an important conceptual step, the guidance remains non-binding and provides no economic incentives. The opportunity costs of this omission are significant: promising device innovations for ultra-rare conditions are delayed or abandoned, and SMEs or academic groups face insurmountable barriers to market entry. By contrast, large manufacturers with broad portfolios can comply with MDR requirements while avoiding high-risk, low-return projects, leaving patients with rare diseases and their clinicians to bear the consequences of foregone innovation. This imbalance underscores the persistent misalignment between the current regulatory architecture and the needs of rare disease populations.

In contrast, the United States (US) established the Humanitarian Use Device (HUD) designation and Humanitarian Device Exemption (HDE) pathway as early as 1990, allowing devices intended for conditions affecting fewer than 8,000 individuals annually to reach the market with reduced evidentiary requirements, albeit under strict usage restrictions and profit conditions (5, 6). While the US framework is not without limitations, it nevertheless provides a clearer and more predictable route to market entry for orphan devices than is currently available in the EU. Notably, the HDE pathway permits marketing approval on the basis of a “probable benefit” standard (requiring a reasonable expectation that the device may provide benefit, even in the absence of definitive evidence of effectiveness) whereas the EU MDR has traditionally required a demonstration of “expected clinical benefit” through comprehensive clinical evaluation. The notion of accepting probable rather than fully demonstrated benefit was absent from European regulatory discourse until the recent MDCG guidance (9, 10).

While regulatory asymmetry explains much of the fragmentation observed in orphan device access, it does not capture the lived realities of those most affected. Patient organizations increasingly act as policy-shaping agents rather than passive beneficiaries, and recent work demonstrates that they contribute to evidence generation, trial feasibility, and strategic prioritization for rare disease technologies (11). Advocacy reports documenting device withdrawal and reimbursement failure describe tangible consequences for patients who rely on implantable systems for motor function or survival, including sudden loss of technological support, disruption of rehabilitation trajectories, and long-term uncertainty for families. These accounts frame orphan device regulation not only as an administrative architecture but as a determinant of continuity-of-care and equity. Integrating patient-centered perspectives therefore strengthens the analytical foundation of this work and clarifies why policy reform must be evaluated in terms of social value, not procedural alignment alone (12, 13).

This paper builds on recent work addressing early access schemes for innovative drugs, but shifts the focus to medical devices, a domain still lacking in legal recognition, policy coherence, and structural support (14). Through a comparative analysis of EU and US approaches to orphan medical devices, we examine the regulatory, clinical, and economic dimensions of early access and market integration. Drawing on the latest guidance, case studies, and policy evaluations, we identify key structural gaps and offer concrete, evidence-based recommendations to ensure that patients with rare diseases are not deprived of vital medical technologies due to regulatory inertia or misaligned incentives. In so doing, we argue for a strategic redesign of orphan device pathways, one that reflects the lessons of orphan drug policy, the realities of device innovation, and the ethical imperative of equitable access.

## 2 Methodology

This study applies a document-based policy analysis to map and compare the regulatory and access pathways for orphan medical devices in EU and the US. The purpose was to identify how regulatory frameworks, evidentiary standards, and incentive structures shape the development and market integration of devices intended for very small patient populations.

We performed a targeted review of academic, regulatory, and legal sources. Peer-reviewed literature published between 1990 and 2025 was retrieved from PubMed and Scopus using focused combinations of keywords such as *orphan device*, *medical device regulation*, *expanded access*, *Europe*, and *United States*. We selected 1990 as the starting point because this was the year in which the United States introduced the Humanitarian Use Device (HUD) designation and Humanitarian Device Exemption (HDE) pathway, establishing the first formal regulatory framework dedicated to orphan medical devices. In parallel, relevant policy and regulatory documents were collected from the European Commission, the Medical Device Coordination Group (MDCG), national competent authorities such as France’s ANSM and Germany’s BfArM, and the US Food and Drug Administration (FDA). Key legislative instruments (namely Regulation (EU) 2017/745 on medical devices (MDR), Regulation (EC) No. 141/2000 on orphan drugs, MDCG 2024–10, and US regulations under 21 CFR Part 814) were examined to capture the legal and procedural foundations of orphan device governance.

Legal commentaries and selected case law were included to contextualize liability and market withdrawal issues, while illustrative case studies were analyzed to highlight real-world regulatory and access challenges. The case studies were selected to reflect different positions within the orphan device ecosystem: (i) Berlin Heart EXCOR illustrates SME-driven development for pediatric use under divergent EU–US evaluation timelines; (ii) Argus II represents a high-profile example of post-market discontinuation and the absence of continuity safeguards for implanted HUD devices; and (iii) the Melody valve demonstrates how a device can progress from humanitarian designation to broader approval when evidentiary generation and reimbursement alignment are feasible. Together, these cases capture heterogeneity in manufacturer size, regulatory trajectory, and patient-level consequences.

Documents were retained if they described mechanisms governing the development, approval, or use of medical devices addressing rare or ultra-rare conditions in the EU or US. Both binding legal texts and informal or *ad hoc* practices were considered. Extracted data were analyzed according to five dimensions: the type of regulatory pathway or designation, its legal basis, evidentiary requirements, associated incentives or constraints, and broader implications for market access and sustainability. Analysis was conducted manually using a structured extraction grid derived from these five dimensions. Each document was screened and charted according to the regulatory pathway considered, its underlying legal basis, the evidentiary requirements applied, the presence or absence of incentives, and the consequences for market access and sustainability. No qualitative coding software was used, as the heterogeneity and legal nature of the documents required interpretive assessment rather than thematic quantification. This approach ensured analytic consistency across sources while preserving the specificity of legislative and policy materials (Table 1).

**Table 1:** Criteria for inclusion and exclusion of documents in the policy analysis.

Category	Inclusion criteria	Exclusion criteria
Population / Scope	Documents addressing medical devices intended for rare or ultra-rare conditions in the EU or US (orphan / humanitarian devices)	Studies focused on devices for common conditions or high-prevalence diseases
Type of document	Regulatory texts, legal documents, policy guidance, regulatory guidance, legal commentaries and case law, health-economic analyses, health technology / access assessments, peer-reviewed academic papers discussing regulatory, reimbursement, access or market-integration aspects	Purely technical, engineering or clinical performance studies without policy / regulatory / access relevance; device-specific performance reports without context
Content relevance	Documents discussing: regulatory pathways; evidentiary requirements; approval or humanitarian designation procedures; access pathways; reimbursement or financing; post-market obligations or exit/discontinuation; liability and withdrawal risks	Articles with no discussion of regulation, access, market entry / exit, reimbursement or structural incentives; purely molecular, laboratory, or technical reports
Language	Publications in English or French (the working languages of the research team and of regulatory communications in EU/US)	
Time frame	Documents published between 1990 and 2025, to encompass the entire evolution from early humanitarian device regulation in the US through the current EU regulatory framework	

Findings were synthesized thematically and comparatively, distinguishing EU-level and national approaches and contrasting them with the US framework to identify structural asymmetries, evidentiary flexibilities, and persistent policy gaps in the governance of orphan medical devices.

This approach carries inherent limitations. Document-based analysis cannot fully capture informal regulatory practices, confidential negotiations, or political considerations that shape real-world implementation. Some access decisions and device discontinuations may involve commercial or institutional factors that are not publicly documented, and therefore remain outside the scope of this review. These constraints do not undermine the validity of the findings, but they indicate that further research, including stakeholder interviews or confidential data access, would complement and deepen the present analysis.

## 3 Regulatory and policy challenges for orphan medical devices

### 3.1 The policy vacuum for orphan medical devices

Over the past two decades, rare disease policy has evolved markedly in the pharmaceutical sector, but its medical device counterpart has remained structurally marginal. The EU’s Orphan Drug Regulation (EC No. 141/2000) has created a robust legal identity for orphan medicines, conferring both market exclusivity and regulatory incentives to stimulate innovation (1, 8, 15). This strategic intervention has led to a significant increase in orphan drug designations and approvals, transforming the treatment landscape for thousands of patients. Yet there exists no parallel legal framework for orphan medical devices in EU: no dedicated designation, no financial incentive system, and no harmonized early access or reimbursement structure (3).

The absence of such a European orphan medical device framework is not merely a bureaucratic omission; it generates a structural inequity manifested in delayed patient access to potentially life-saving technologies and in reduced incentives for innovation, particularly for SMEs and academic developers. Medical devices play a critical role in rare disease care: from diagnostic instruments and assistive technologies to implantable interventions where no pharmacological alternative exists. For example, pediatric ventricular assist devices (VADs) and customized spinal implants are often the only viable therapeutic options in specific clinical contexts (4). Yet the development of these devices remains largely unsupported by the mechanisms that have catalyzed orphan drug innovation. Without designated regulatory routes, device developers face full conformity assessment obligations under the MDR, often with small patient cohorts, limited trial feasibility, and no guarantee of reimbursement upon approval.

The US, while not immune to similar challenges, has at least provided a partial solution. The Humanitarian Use Device (HUD) designation and associated Humanitarian Device Exemption (HDE) pathway were introduced under the Safe Medical Devices Act of 1990, establishing a legal and regulatory route for devices intended to benefit ≤8,000 individuals annually in the US (5, 6). Unlike standard Premarket Approval (PMA), the HDE pathway allows for marketing based on a demonstration of “probable benefit” without requiring full evidence of efficacy, a pragmatic concession to the data constraints inherent in rare disease settings (10). Although the HDE program includes limitations (most notably profit restrictions unless the device is for pediatric use as an economic incentive to facilitate device development), it nonetheless formalizes a lower evidentiary threshold and enables legitimate, Institutional Review Board (IRB)-supervised access for patients.

By contrast, the EU’s response to the unique challenges of orphan devices has remained diffuse. Until recently, rare-use medical devices could only be accessed through legal exceptions—most notably Article 59 of the MDR—which allows Member States to temporarily authorize non-CE-marked devices in the interest of public health or individual patient need. However, Article 59 was not designed as an orphan-specific mechanism; it is reactive by nature, used mostly in emergencies (e.g., COVID-19 ventilator shortages), and does not confer any marketing status or long-term reimbursement guarantee (9). Custom-made devices under Article 5(5) of the MDR from 2017 offer another path for unique cases, but they are exempt from CE-marking only for single-patient prescriptions, making them inherently limited to one-off use and therefore non-scalable at the population level, far more restrictive than the US HDE threshold of ≤ 8,000 patients per year.

The release of the MDCG 2024–10 guidance marks the first coordinated attempt to articulate an EU-level definition and evaluation standard for orphan medical devices. The document defines an orphan device as one intended for a disease or condition affecting no more than 12,000 individuals per year across the EU, and where either insufficient alternatives exist or the device offers a clear expected clinical benefit (9). This numerical threshold mirrors the FDA’s 8,000-person definition when scaled to the EU population, acknowledging the transatlantic convergence in epidemiological rationale. The guidance also introduces key flexibilities: it permits market approval with limited pre-market clinical data under strict post-market obligations and encourages the use of extrapolated evidence or off-label legacy data where justified. Yet, despite these advances, the MDCG guidance remains a soft-law instrument, endorsed but not legally binding, and without embedded financial or regulatory incentives.

### 3.2 Divergent regulatory pathways in the EU and US

The regulatory architecture governing medical device approval differs markedly between the EU and the US: not only in structure and centralization, but in how each system conceptualizes early access, evidentiary flexibility, and the ethical obligations in relation to rare disease innovation. While both regions have introduced mechanisms to manage uncertainty in high-risk technologies, only the US has formalized a pathway for orphan devices that balances regulatory pragmatism with patient protection. In the EU, despite recent interpretive guidance, orphan devices remain structurally embedded within a framework that was never designed to accommodate extreme scarcity of evidence, patients, or commercial viability.

#### 3.2.1 The European Union framework

Under the EU’s Medical Devices Regulation (EU 2017/745), all medical devices, regardless of novelty, target population, or therapeutic urgency, are subject to a uniform system of conformity assessment. Devices are classified into four risk-based categories (Class I to III and active implantables), with increasing requirements for clinical evidence, post-market surveillance, and oversight by notified bodies (7). For Class IIb and III devices, where orphan technologies are typically concentrated, the burden of proof for safety and clinical performance is substantial, even when traditional randomized controlled trials are unfeasible due to patient scarcity. The MDR introduced a more rigorous interpretation of the General Safety and Performance Requirements (GSPRs), thereby raising the bar for market access relative to prior directives. This shift has been further clarified in subsequent MDCG guidance (9).

Importantly, the MDR does not distinguish between common and rare conditions. It contains no statutory definition of orphan devices, no mechanism for reduced evidentiary thresholds based on disease prevalence, and no provisions for economic incentives such as fee reductions, market exclusivity, or public funding for evidence generation. Instead, two exceptions serve as de facto early access routes: Article 59, which permits temporary national derogations for non-CE-marked devices in the interest of public health or to meet the specific needs of individual patients, and Article 5(5), which allows in-house manufacturing by healthcare institutions under narrowly defined conditions. Neither was designed for rare disease contexts.

This omission reflects deep institutional path dependence: the medical device regulatory regime in the EU has long centered on conformity assessment and risk classes rather than on strategic differentiation by disease rarity. Moreover, the political economy of the device sector further entrenches this status quo: the industry is highly fragmented, dominated by SMEs (representing approximately 90 percent of companies in the EU), which often lack the resources or collective capacity to lobby for orphan-device provisions (16). Only a small subset of these SMEs appear to engage in orphan-device development, as suggested by MDCG stakeholder consultations and parliamentary hearing records reporting limited continuity beyond prototyping or early feasibility stages. Emerging post-MDR transition reports indicate that recertification failure and voluntary market withdrawal disproportionately affect low-volume devices, particularly those without commercial investors or a secured reimbursement pathway (16). The absence of centralized advocacy mechanisms further constrains SME influence over regulatory reform, in contrast with highly coordinated pharmaceutical lobbying structures. Finally, fiscal caution may disincentivize regulators from extending orphan-style incentives to devices, given concerns about budgetary exposure: unlike pharmaceuticals, devices are frequently viewed as capital goods or hospital-level tools, making payers more reluctant to introduce exclusivities or financial stimulus instruments.

#### 3.2.2 Emerging European guidance: MDCG 2024–10

This regulatory vacuum was partially addressed in June 2024, when the Medical Device Coordination Group published MDCG 2024–10, a guidance document providing the first EU-level definition of orphan medical devices (9). According to this guidance, a device may qualify as “orphan” if it is intended for a condition affecting no more than 12,000 individuals per year across the EU, and either (1) no adequate alternatives exist, or (2) the device offers a meaningful clinical benefit over existing standards of care. The document further clarifies that, under justified conditions, limitations in pre-market clinical evidence may be acceptable, provided that manufacturers commit to rigorous post-market clinical follow-up (PMCF), transparent communication with users, and early engagement with expert panels (9). Yet the guidance is not legally binding. It does not override the MDR’s core obligations, and it lacks the force of a Directive or Regulation. Nor does it offer economic incentives such as fee reductions, market exclusivity, or public funding for evidence generation.

#### 3.2.3 The United States framework

In contrast, the US has institutionalized a more defined and legally grounded pathway. The Humanitarian Use Device (HUD) and Humanitarian Device Exemption (HDE) framework, established under the Safe Medical Devices Act of 1990 and refined by the 21st Century Cures Act of 2016, creates a designated route for medical devices intended to benefit ≤8,000 individuals annually (5, 6). Upon designation as a HUD, a device becomes eligible for HDE approval, which allows marketing based on a demonstration of “probable benefit.” This differs from the effectiveness requirements of Sections 514 and 515 of the Federal Food, Drug, and Cosmetic Act (FD&C Act). This evidentiary flexibility acknowledges the difficulty of generating randomized data in ultra-rare populations and provides a structured, legally recognized mechanism for early access (10).

The HDE pathway also imposes important safeguards. Use of the device must occur under Institutional Review Board (IRB) supervision, and profit margins are restricted for devices used in pediatric populations, to incentivize the development of such highly needed devices in this market segment. An annual distribution number (ADN), originally set at 4,000 and later expanded to 8,000, limits the commercial scope of the device’s application (5, 6, 17). Under the HDE framework, manufacturers may not sell devices for profit unless they target a pediatric population, and annual distribution is capped at an ADN pursuant to FD&C Act §520(m) and 21 CFR 814 Subpart H (18). While these constraints have been criticized for limiting financial viability, they also reflect a clear policy trade-off. Regulators enable accelerated access by relaxing evidentiary demands. In return, they cap commercial incentives to reduce overpromotion and inappropriate use.

#### 3.2.4 Comparative analysis: codification versus interpretation

Perhaps most critically, the HUD/HDE framework represents more than a technical pathway, it signals the regulatory system’s recognition that rare disease devices require tailored rules. By establishing orphan status in law, the US FDA has created an institutional point of entry for sponsors, clinicians, and payers to navigate the development and integration of high-need, low-volume technologies. This clarity is largely absent in the EU, where manufacturers must navigate an opaque combination of general conformity rules, exceptional derogations, and discretionary guidance, often without assurance of reimbursement or clinical uptake. The contrast also reflects a broader regulatory philosophy: while the US system tends to prioritize timeliness and pragmatic access in areas of high unmet need, the EU traditionally favors procedural rigor and more extensive evidence accumulation before conferring new designations (10, 19). This pattern mirrors the slower evolution of the Orphan Drug Regulation itself, where incentives for rare diseases emerged later and were more narrowly framed than in the US (3). Together, these differences have produced a structurally more conservative and risk-averse environment for orphan devices in EU, where flexibility often arises from interpretation rather than codification (20).

The divergent philosophies of these two systems (codification versus interpretation, structural designation versus *ad hoc* exception) have direct consequences for patients and developers alike. In the US, the existence of a statutory orphan-device pathway enables strategic planning, early engagement with regulators, and alignment of development trajectories with the specific needs of small patient populations. In EU, by contrast, even the most clinically essential devices may face regulatory inertia, fragmented national interpretations, or withdrawal due to non-viability under the MDR’s recertification regime (4).

Taken together, these contrasts reveal a transatlantic divergence in regulatory philosophy: the US codifies flexibility, whereas the EU interprets it. Both systems pursue patient safety and evidentiary integrity, yet they embody opposing logics of innovation. The US embeds rare-disease pragmatism within statutory law, offering predictable access routes and institutional coherence. The EU, by contrast, relies on interpretive flexibility within a rigid conformity regime: producing fragmented access, delayed innovation, and weaker economic viability for orphan devices (Table 2).

**Table 2:** Comparative overview of orphan medical device regulation in the EU and the US.

Dimension	European Union (EU MDR 2017/745)	United States (HUD/HDE framework)
Legal status	No formal orphan-device designation	Legally codified orphan-device category (≤8,000/year)
Evidentiary standard	“Expected clinical benefit” (full CE-marking requirements)	“Probable benefit” (reduced evidentiary threshold)
Flexibility mechanism	Exceptional derogations (Art. 59, Art. 5(5)); non-binding MDCG 2024–10 guidance	Statutory humanitarian pathway under the Safe Medical Devices Act
Financial incentives	None (no fee waivers, no exclusivity)	Profit permitted for pediatric uses; annual distribution cap
Oversight	Decentralized (Notified Bodies, national authorities)	Centralized FDA oversight with IRB supervision
Policy philosophy	Interpretive flexibility within rigid law	Codified flexibility balancing access and safeguards
Systemic consequence	Fragmented access, limited viability	Predictable early access, structured incentives

### 3.3 Structural barriers to access and innovation

Despite growing recognition of their therapeutic importance, orphan medical devices face a unique convergence of structural barriers that impede their development, evaluation, and integration into clinical care. These barriers are not merely technical but systemic, arising from a misalignment between regulatory expectations, economic feasibility, and evidentiary realities in ultra-rare populations. In both the EU and the US, the result is a persistent fragility in the ecosystem for device-based innovation in rare diseases, a fragility that undermines both patient access and public health equity.

The first and most fundamental barrier is evidentiary. Unlike pharmaceutical interventions, which benefit from decades of methodological standardization and multicenter trial infrastructure, the clinical evaluation of medical devices (especially in rare conditions) remains deeply heterogeneous. Device efficacy often depends on context-sensitive variables: surgical technique, operator expertise, learning curves, and institutional environments (21). These challenges are magnified in orphan indications, where randomized controlled trials are often infeasible due to extremely small populations and ethical constraints on withholding potential benefit. Consequently, developers must rely on alternative sources of evidence: retrospective case series, real-world data, registries, or historical controls. Yet such approaches, while methodologically defensible in rare contexts, are often deemed insufficient under the high bar set by the EU’s MDR or the FDA’s standard PMA process (19).

The recent MDCG 2024–10 guidance in the EU represents an important shift in this regard. It explicitly acknowledges that, for orphan devices, “limitations in pre-market clinical data may be acceptable,” provided that the benefit–risk profile is justified, and post-market follow-up is rigorously implemented (9). Similarly, the US HDE pathway requires only “probable benefit” rather than full effectiveness. However, these regulatory concessions do not erase the operational burden of evidence generation. In the EU, orphan device sponsors are expected to enroll the majority (ideally more than 90%) of all eligible patients in structured post-market clinical follow-up (PMCF) studies or registries (9). In practice, this requires sophisticated data infrastructure, multi-center coordination, and significant financial and logistical capacity, resources that are often beyond the reach of small and mid-sized developers.

Economic viability constitutes the second major barrier. Unlike orphan drugs, which in the EU benefit from 10-year market exclusivity and a range of fiscal incentives, orphan medical devices lack any formal protection or financial support (2). The cost of bringing a high-risk device to market (particularly under the expanded conformity assessment requirements of the MDR) can exceed €10 million, a figure largely disproportionate to the limited market size of rare disease indications. By contrast, while development costs for orphan drugs have been estimated to reach several hundred million euros, their lifetime revenues often exceed 1 billion euros due to exclusivity incentives, premium pricing, and global market reach (22–24). This cost–benefit asymmetry makes the structural imbalance between drug and device incentives particularly stark: whereas orphan drugs benefit from predictable economic returns enabled by regulatory and fiscal advantages, orphan device developers face high upfront costs with limited prospects for return on investment. In the US, the HUD/HDE framework offers a reduced evidentiary threshold but includes profit restrictions: devices approved under HDE cannot be sold for profit unless they address a pediatric population, and their distribution is capped by an annual limit (5, 6). While this cap was raised from 4,000 to 8,000 units by the 21st Century Cures Act, the underlying tension remains: developers are expected to invest in clinical trials, regulatory compliance, and manufacturing despite having no realistic prospect of recouping their costs.

The lack of tailored reimbursement pathways further compounds this dilemma. In the EU, reimbursement decisions are made at the Member State level, often through health technology assessment (HTA) bodies that apply evidence standards calibrated for common diseases and pharmaceuticals. As a result, devices serving ultra-small populations are systematically undervalued, as traditional incremental cost-effectiveness ratios (ICERs) fail to capture broader innovation or societal value (15, 25). Even CE-marked orphan devices may face long delays or outright rejection in national reimbursement schemes due to perceived data insufficiency or uncertain cost-effectiveness (21). In France, innovative devices may be temporarily covered under programs such as the *Innovation Funding* or the transitional *Prise en charge transitoire* (PECT) scheme, but these remain exceptions rather than the rule (26). In the US, HDE approval does not guarantee coverage by public or private payers. While the Centers for Medicare and Medicaid Services (CMS) may issue national or local coverage determinations, these are rare and inconsistently applied. The proposed Medicare Coverage of Innovative Technologies (MCIT) rule, which could have streamlined coverage for FDA-designated breakthrough devices, was ultimately repealed in 2021, leaving no systematic reimbursement route even for devices targeting a high unmet need (27).

Beyond reimbursement, intellectual property (IP) protection poses an additional barrier to innovation. Securing patents for medical devices is often more challenging than for pharmaceuticals, as regulatory standards require demonstrable safety and a clear “technical effect” (28, 29). This complexity intensifies for devices incorporating software or artificial intelligence (AI): under European patent law, such systems must explicitly demonstrate how algorithms improve clinical outcomes or device performance to qualify for protection (EPO, 2023b). For example, an AI-enabled heart monitor must show measurable gains in diagnostic accuracy or patient safety to meet eligibility criteria (28, 29). These constraints further deter small developers who already face high regulatory costs and uncertain reimbursement prospects (30).

Finally, the risk of supply discontinuation threatens continuity of care for patients already dependent on orphan devices. In the EU, the MDR’s recertification requirements have led to market withdrawals of legacy devices, including those used in pediatric cardiology and neuromuscular care (4). These losses are often invisible to policymakers, but acutely felt by clinicians and patients. As of 2024, the MDR includes a provision requiring 6 months’ notice before a device is withdrawn, but no formal mechanism exists to ensure continuity, replacement, or market re-entry.

In the US, there is similarly no statutory obligation for continued supply of HDE devices once they become commercially unviable. This creates what contract theorists describe as a *hold-up problem*: patients and clinicians become locked into a technology on which they depend for daily functioning, yet manufacturers retain unilateral exit rights when profitability declines (31). The case of the Argus II retinal prosthesis illustrates this asymmetry. Production and development of the device were discontinued in 2020, leaving implanted patients temporarily without access to maintenance or technical assistance. In August 2023, Second Sight Medical Products merged with Nano Precision Medical to form Vivani Medical, announcing a renewed commitment to provide limited technical support for existing Argus II users (32, 33).

These structural barriers (evidentiary, economic, and institutional) are not merely technical inefficiencies but components of a self-reinforcing cycle. Limited evidence weakens reimbursement prospects, weak reimbursement undermines commercial viability, and reduced commercial viability discourages further evidence generation, thereby reproducing the initial data deficit. This dynamic reflects a deeper policy failure to recognize medical devices as integral components of rare disease strategy. In both the EU and the US, fragmentation between regulatory approval, reimbursement, and long-term access leaves even clinically essential orphan devices vulnerable to underdevelopment, underutilization, or disappearance. Bridging this gap requires more than regulatory flexibility; it demands a rethinking of policy priorities, economic models, and ethical obligations in the governance of rare disease technologies.

Table 3 highlights this asymmetry starkly: in the US, scarcity is codified through a defined HUD/HDE designation, embedding flexibility into statute, whereas in the EU, rarity is managed by exception within a conformity-based regime. This distinction shapes incentives but neither model delivers a coherent pathway from designation to sustained access. As a result, the very populations for whom innovation is most urgent remain the least structurally protected.

**Table 3:** Comparative overview of orphan medical device frameworks: EU vs. US.

Dimension	EU (EU MDR 2017/745)	US (HUD/HDE framework)
Legal orphan designation	No. Defined only in *MDCG 2024–10* (non-binding guidance).	Yes. *Humanitarian Use Device (HUD)* designation, legally defined since 1990.
Prevalence threshold	≤ 12,000 individuals/year across the EU (*guidance only*). Definition differs from orphan-drug criteria (≤ 5 in 10,000) and from US device thresholds, creating cross-sector inconsistencies.	≤ 8,000 individuals/year in the US (*statutory*). Threshold distinct from the US orphan-drug definition (≤ 200,000 patients), highlighting the absence of a uniform standard across product types.
Regulatory pathway	MDR conformity assessment; possible Article 59 derogation (case-by-case, no market status).	*Humanitarian Device Exemption (HDE)* pathway.
Evidentiary standard	“Expected clinical benefit” + PMCF; allows extrapolation (*MDCG 2024*).	“Probable benefit” without full demonstration of effectiveness.
Post-market obligations	High: ≥ 90% of eligible patients in PMCF recommended.	Required: IRB oversight and annual reporting to FDA.
Economic incentives	Limited and structurally insufficient. Orphan medical devices may rely on intellectual property rights (IPRs), yet securing and maintaining patents is challenging due to stringent regulatory standards, software–hardware updates, and exclusions under Art. 53(c) EPC (28). In the absence of a legal orphan-device framework, no complementary incentives (such as data exclusivity, fee waivers, or market protection) exist (30, 34)	Restricted incentives. IPRs available, but profit prohibited except for pediatric uses; capped annual distribution (8,000 units). HDE status nonetheless provides a legal route with predictable evidentiary flexibility and limited commercial feasibility.
Reimbursement pathway	National-level, heterogeneous; no EU-wide orphan-specific HTA framework.	Case-by-case; no automatic CMS coverage for HDE devices.
Supply continuity measures	Six-month notice before withdrawal (MDR Art. 123); no obligation to maintain availability.	No obligation to continue supply once commercially non-viable.
Binding legal status	Soft law (guidance); no statutory recognition of orphan status.	Binding statute under *Safe Medical Devices Act*.
Integration with rare-disease strategy	Fragmented; not aligned with EU rare-disease plans or drug incentives.	Partial alignment; HUD/HDE recognized in rare-disease policy but not fully integrated.
Alignment of incentives for small vs large firms	Structure disproportionately favors large diversified manufacturers able to absorb compliance and IP costs. SMEs (the main innovators in rare-disease devices) face prohibitive regulatory and financial barriers.	More balanced: centralized FDA guidance and predictable criteria allow smaller innovators to engage earlier, though profit restrictions still limit investment sustainability.

## 4 Case study: orphan devices at the margins of innovation

The abstract category of “orphan medical devices” becomes tangible when examined through concrete trajectories that expose structural patterns rather than isolated mishaps. Across the EU and US, multiple innovations designed for rare conditions show how regulatory ambiguity, reimbursement fragmentation, and the absence of tailored economic incentives converge to jeopardize timely access and post-market continuity, even where clinical value is clear. The cases below are organized to make explicit the EU–US contrast (approval timing, legal pathway, post-market safeguards) and the industrial lens (SMEs versus large firms).

### 4.1 Pediatric ventricular assist devices (VADs) in end-stage heart failure: *Berlin heart EXCOR*

*Regulatory timing and pathway*: Developed in Germany by an SME, the EXCOR was in clinical use in EU since the 1990s and available via CE marking well before the US. In the US, an HDE (Humanitarian Device Exemption) was granted on 16 December 2011 on the basis of *probable benefit* from a multicenter prospective study in 48 children; this was the first HDE for a pediatric VAD. Subsequently, once longer-term effectiveness evidence accrued, the device obtained PMA (premarket approval) in 2017.

*EU recertification and continuity risks*: Under the MDR (EU 2017/745), recertification costs and notified-body capacity have made the pathway increasingly burdensome for legacy pediatric devices, with reports of delays that constrain access despite the absence of alternatives. This illustrates a continuity-of-care vulnerability in the EU: even clinically indispensable orphan devices can be threatened by procedural inertia during regime transitions (4).

*Industrial implication*: An SME can pioneer a life-saving niche technology yet struggle to maintain certifications and supply on a minuscule market without dedicated incentives (fee relief, supported evidence generation, continuity safeguards). In the US, the HDE allowed profit for specified pediatric uses, which partially mitigated viability constraints; the EU lacks analogous, codified orphan-device incentives.

### 4.2 Retinal prosthesis for retinitis pigmentosa: a*rgus II*

*Regulatory timing and pathway:* The CE mark was granted March 2011 (EU), followed by FDA HDE approval in February 2013 (US) on a *probable benefit* standard. Thus, EU approved first, the US second with formal humanitarian safeguards (IRB oversight, post-market obligations).

*Commercial exit and time-inconsistency*: Facing limited sales and fragmented reimbursement, the manufacturer ceased production (2019) and massively downsized in March 2020, leaving ~300–350 implanted patients worldwide without maintenance or software updates. Between 2020 and 2023, there was no assured support, a textbook time-inconsistency (policymakers approve access ex ante without embedding ex post safeguards) and a hold-up problem (patients locked into an implant; the firm retains unilateral exit rights).

*Partial remediation*: After a corporate transaction in 2023, limited technical support resumed (replacement external components from remaining stock; upgrade path via the *Argus 2 s* external system cleared in 2021). The window of unsupported years nonetheless reveals a regulatory lacuna on continuity obligations for implanted orphan devices in both jurisdictions.

### 4.3 Transcatheter pulmonary valve for a rare congenital lesion: *Medtronic melody*

*Regulatory timing and pathway:* The Melody valve received CE marking in October 2006 (EU) following early French clinical experience, offering a minimally invasive solution for children with right-ventricular outflow tract conduit dysfunction. In the US, the FDA granted an HDE in January 2010, limiting use to ≤ 4,000 patients per year and requiring extensive five-year post-approval studies. After evidence of durability and procedural safety accumulated, the FDA issued full PMA approval in January 2015, expanding indications and lifting humanitarian restrictions.

*Regulatory and economic implications:* The case demonstrates how large manufacturers can navigate the US humanitarian pathway when financial parameters are predictable. Medtronic leveraged the 2007 profit allowance for pediatric HDE devices, enabling sustainable pricing within the humanitarian cap while financing required surveillance. In EU, earlier CE entry offered a four-year access advantage, but diffusion depended on country-by-country reimbursement decisions and the capacity of expert centers. The Melody’s trajectory suggests that when regulatory rules combine clear evidentiary expectations with bounded commercial feasibility, large firms can bring orphan devices from accelerated entry to full approval without jeopardizing continuity, an outcome far less attainable for SMEs operating under the EU’s fragmented system.

### 4.4 Patient-specific 3D-printed implants for craniofacial anomalies

*Regulatory fit and access*: Additive manufacturing enables bespoke implants for children with rare craniofacial syndromes where standard devices are unusable. Yet one-off production and small N populations make classical trials impracticable. In the EU, such implants are typically produced under Article 5(5) (MDR in-house manufacture) or Article 59 derogations, with no formal orphan-device status, limited reimbursement, and unequal access restricted to specialized centers.

*Systemic implications*: The absence of a harmonized pathway leaves only highly resourced academic hospitals able to design and deliver such implants, reinforcing geographic and socioeconomic disparities. In the US, comparable products fall under custom-device exemptions within FDA’s additive-manufacturing guidance (2017), which allows flexibility but demands rigorous traceability and quality documentation. Both frameworks treat individualized implants as regulatory exceptions rather than integral components of modern precision medicine. This structural misalignment (technical feasibility without institutional scalability) illustrates how orphan-level innovation risks remaining confined to elite settings absent a dedicated adaptive framework (Table 4).

**Table 4:** Cross-case synthesis: structural contrasts between the EU and US frameworks for orphan medical devices.

Dimension	European Union (EU)	United States (US)	Analytical implication
Approval timing	CE marking typically granted earlier (e.g., *Argus II*, 2011; *Melody*, 2006), often based on limited early data.	HDE approvals granted later (e.g., *Argus II*, 2013; *Melody*, 2010; *EXCOR*, 2011) but under a codified humanitarian pathway.	Europe offers *faster entry* but without legal orphan status; the US provides *structured flexibility* through law, though with initial delays.
Standard of proof	“Expected clinical benefit” and PMCF; no defined orphan-device threshold.	“Probable benefit” standard under HDE, with mandatory post-approval studies and IRB oversight.	US approach formalizes evidentiary pragmatism; EU relies on case-by-case interpretation, producing uncertainty for developers.
Economic incentives	No orphan-specific financial instruments; SMEs face high MDR recertification costs.	Profit prohibited under HDE except for pediatric indications (post-2007 amendment), improving viability for niche devices.	US framework includes *bounded commercial feasibility*; EU offers none, disincentivizing innovation among SMEs.
Continuity safeguards	No binding mechanism for continued supply or service; six-month withdrawal notice under MDR Art. 123.	No statutory obligation to ensure post-market continuity once a device becomes commercially nonviable.	Both systems lack *ex post*stewardship obligations; Argus II illustrates **time-inconsistency** and **hold-up** risk.
Reimbursement landscape	Fragmented national HTA and payer assessments; no EU-wide orphan-device coverage model.	CMS or private-payer coverage case-by-case; no automatic linkage to FDA approval.	In both systems, regulatory clearance ≠ market access; policy disconnect persists between evidence and payment.
Industrial structure	Sector dominated by SMEs (92%), limited lobbying power, vulnerable to regulatory cost shocks.	Larger diversified firms can cross-subsidize niche products; SMEs remain fragile but benefit from clearer FDA interlocution.	The EU framework disproportionately penalizes SMEs; the US model offers predictability but not profitability.
Integration with rare-disease policy	Orphan devices not integrated into EU rare-disease plans or incentives under Regulation (EC) 141/2000.	HUD/HDE partially recognized within US rare-disease strategy but without reimbursement alignment.	Absence of a full continuum from designation → reimbursement → continuity undermines patient protection.
Ethical and systemic effect	Flexible ex ante (approval) but fragile ex post (continuity).	Structured ex ante (law) but incomplete ex post (coverage).	**Structural asymmetry:** EU codifies speed; US codifies safety. Neither ensures sustainability for patients dependent on orphan devices.

## 5 Policy recommendations

The structural neglect of orphan medical devices in both the EU and the US is not merely an oversight, it is a policy failure. It stems from an outdated dichotomy that treats drugs as the primary vector of innovation, particularly in rare diseases, and devices as peripheral, even when the latter are indispensable for diagnosis, therapeutic delivery, monitoring, or functional assessment. Based on the evidence presented, we propose five core recommendations to align policy with clinical and ethical reality.

### 5.1 Establish a legal designation for orphan medical devices in the EU

The EU urgently needs a binding legal definition of “orphan medical device,” similar to the HUD framework in the US. This designation should be codified through a delegated act or legislative amendment and linked to specific prevalence thresholds (e.g., ≤12,000 patients/year across the EU, per MDCG, 2024). Without formal recognition, developers cannot benefit from regulatory flexibility, targeted funding, or coordinated reimbursement dialog.

### 5.2 Introduce tailored regulatory pathways with adaptive evidentiary requirements

Regulators must operationalize proportionality in evidence generation. Building on MDCG 2024–10 and FDA’s “probable benefit” threshold, the EU should implement a specialized conformity assessment track for orphan devices that permits robust real-world evidence, single-arm trials, or historical control data as acceptable bases for approval, particularly when RCTs are infeasible (19, 21). This pathway should integrate post-market data collection with clear guidance and oversight to ensure effective management and control.

### 5.3 Create sustainable economic incentives beyond approval

Approval alone does not ensure availability. Once an orphan device reaches the market, sustainability depends less on intellectual property protection (which already provides baseline exclusivity) than on mechanisms that offset the structural diseconomies of small patient populations. In the EU, manufacturers should benefit from targeted financial instruments, including partial fee waivers for notified-body assessments, dedicated funding for PMCF infrastructure, and EU-level pooled procurement or volume-guarantee schemes for ultra-rare indications (MDR 2017/745, Annex XIV, Part B). These mechanisms would offer predictable demand and de-risk production, particularly for SMEs that cannot absorb the costs of maintaining certification and post-market surveillance across multiple jurisdictions.

In the US, the current prohibition on profit for HDE devices (except pediatric) should be revisited to allow limited, performance-based returns under conditions of high unmet need and continuous evidence generation (3, 15). Comparative economic modeling contrasting, for instance, five-year pooled procurement guarantees with temporary exclusivity extensions could help identify cost-effective strategies to sustain orphan-device supply while preserving affordability and public trust.

### 5.4 Integrate reimbursement and early access pathways at national and EU level

HTA agencies must adapt their assessment frameworks to the specificities of orphan devices. This includes accepting context-sensitive evidence, evaluating non-traditional endpoints (e.g., cognitive function in non-verbal children), and applying ethical and equity-based criteria. At the EU level, a centralized early access mechanism for orphan devices (analogous to EMA’s PRIME for medicines) could support temporary coverage and data generation. National programs, like France’s Innovation Funding or Germany’s §137e pathway, offer models worth scaling and harmonizing.

### 5.5 Mandate continuity of access to device support for medical implants

Post-approval abandonment of orphan devices represents a major ethical and policy failure. Regulatory authorities should require sponsors to submit continuity-of-access plans as a condition of approval, particularly for implantable or life-sustaining technologies. These plans should specify minimum service durations, spare-part supply, and pathways for technology transfer or licensing in the event of market exit.

To operationalize this safeguard, authorities could establish a form of mandatory “exit insurance,” whereby firms either pre-fund long-term patient support or contribute to a pooled continuity fund covering maintenance and technical assistance if a manufacturer withdraws. Such mechanisms would align incentives, internalize long-term responsibility, and prevent a recurrence of cases like the Argus II retinal implant, where patients were left unsupported for years following the company’s collapse (33). Public co-financing could complement this model in extreme circumstances to ensure ethical continuity of care.

Collectively, these proposals are best understood as a staged reform rather than a parallel set of interventions. The first requirement is legal recognition of orphan devices, since no incentive scheme, evidentiary adaptation, reimbursement mechanism or supply guarantee can operate without a formal category to anchor them. Once this foundation exists, regulatory flexibility, economic stimuli, HTA alignment and continuity protections become not only feasible but mutually reinforcing. Prioritization therefore lies not in importance but in sequence: definition enables incentives, incentives enable evidence generation, and evidence sustains access over time.

## 6 Conclusion

The persistent absence of a coherent policy framework for orphan medical devices is not a marginal gap; it is a structural contradiction at the core of contemporary rare disease policy. As therapeutic innovation becomes increasingly personalized, data-driven, and technologically integrated, regulation must evolve to prevent a new form of inequity, one in which the availability of sophisticated drugs outpaces the tools needed to diagnose, monitor, and sustain their effects.

Unlike orphan drugs, which enjoy clear legal status and layered financial incentives, orphan devices operate within a vacuum of recognition and sustainability. This vacuum is not neutral. It systematically disadvantages the most vulnerable technologies: those serving small, heterogeneous patient populations, where clinical evidence is harder to generate and commercial viability is structurally limited.

What is required now is not merely regulatory refinement but an inflection point equivalent to the 1983 US Orphan Drug Act, a moment that redefines how society values innovation serving the few. Just as that legislation transformed the economics of rare disease drug development, a comparable framework is now needed for devices to unlock investment, ensure continuity, and embed equity into the architecture of technological innovation. The worth of an intervention should no longer be measured by its market size or trial scale, but by its necessity, its societal contribution, and its capacity to restore function or dignity in rare conditions.

The analyses presented in this paper demonstrate that the failure is systemic rather than exceptional. The reforms proposed (legal recognition, adaptive evidentiary standards, targeted economic incentives, HTA integration, and mandatory continuity plans) are pragmatic responses to this systemic neglect.

Ultimately, the question is not whether orphan devices deserve exceptional treatment, but whether patients with rare conditions deserve consistent and equitable access to the full continuum of innovation. To act on this is to honor the founding principle of rare disease policy: that rarity should never determine the quality of care.
